# Quality Indicators for Evaluation of Neonatal Basic Care in Nutrition and Feeding: A Scoping Review and Modified Delphi Study

**DOI:** 10.3390/nu18193230

**Published:** 2026-09-30

**Authors:** Pablo Buck Sainz-Rozas, Dimelza Osorio Sánchez, Pablo García Molina, Evelin Balaguer-López, Sergio Alonso Fernández

**Affiliations:** 1 Health Services Research Group, Vall d’Hebron Institut de Recerca (VHIR), 08035 Barcelona, Spain; 2 Methodology of Biomedical Research and Public Health, Universitat Autònoma de Barcelona (UAB), 08193 Bellaterra, Spain; 3 Centro de Investigación Biomédica en Red de Epidemiología y Salud Pública (CIBERESP), Instituto de Salud Carlos III, 28029 Madrid, Spain; 4 Hospital Clínico Universitario de Valencia, 46010 Valencia, Spain; 5 Department of Nursing, Universitat de València, 46010 Valencia, Spain; 6 Fundamental and Clinical Nursing Department, Nursing Faculty, University of Barcelona (UB), 08907 Barcelona, Spain; 7 Nursing Research Group (GRIN), Bellvitge Biomedical Research Institute (IDIBELL), 08908 L’Hospitalet de Llobregat, Spain

**Keywords:** breastfeeding, neonatal nutrition, neonatal nursing, nursing-sensitive indicators, Delphi method, quality indicators, RAND/UCLA, patient safety

## Abstract

**Background/Objectives:** Despite the clinical relevance of nutrition and feeding in neonatal intensive care, no consensus-based set of quality indicators currently exists to systematically evaluate basic nutritional care in hospitalised neonates. This study aimed to identify, agree through expert consensus and prioritise a structured set of nursing-sensitive quality indicators for this domain. **Methods:** A two-phase methodological study combined a Scoping Review (Joanna Briggs Institute methodology; prospectively registered on OSF; reported per PRISMA-ScR) and a two-round modified Delphi study based on the RAND/UCLA Appropriateness Method. Four databases (PubMed, Embase, Scopus, and Cochrane Library; 2020–2025) were searched, complemented by snowball and the grey literature. Twenty-seven candidate indicators synthesised inductively into eight domains were rated by 23 expert nurses across three dimensions (appropriateness, clinical validity, and feasibility) on a nine-point Likert scale. **Results:** Retention from Round 1 to Round 2 was 87% (*n* = 20). Twenty-two indicators (81.5%) were classified as appropriate and five (18.5%) as uncertain, in all cases because of feasibility. The overall median for appropriateness and clinical validity was 9.0 in both rounds. Feasibility showed greater variability, with an 18% mean IQR reduction after Round 2. Indicators reaching consensus were distributed across structure (*n* = 3, 13.6%), process (*n* = 16, 72.7%) and outcome (*n* = 3, 13.6%) dimensions. An exploratory post-hoc prioritisation analysis identified four implementation tiers; the five Tier 1 indicators ranked in the top five under every weighting scheme tested. **Conclusions:** This consensus-based set provides a structured basis for monitoring neonatal nutritional care quality and for reducing clinical variability. Validation by a broader interdisciplinary panel including families, and prospective testing in routine practice, are required before its use in clinical dashboards or benchmarking.

## 1. Introduction

Approximately 17 million preterm births occur globally each year, making prematurity the most frequent condition of the neonatal period and a major global public health concern [1]. Neonatal care faces a high burden of morbidity and mortality. Although neonatal survival has improved markedly over recent decades, the heterogeneous and often unmeasured quality of the care that determines long-term outcomes have exposed a parallel challenge [2,3,4].

The improvements observed in survival have been driven not only by technological advances but also by the consolidation of organisational and clinical strategies grounded in evidence [5,6]. Developmental and family-centred care models have transformed neonatal care and are associated with improved clinical outcomes [2,7,8]. Within these models, basic neonatal care—the essential interventions aimed at ensuring stability, development and safety of the hospitalised newborn and their family—has emerged as a structuring axis of quality [9,10].

The Donabedian model provides a widely accepted framework for assessing healthcare quality. However, its operationalisation into specific, validated indicators has remained heterogeneous in the neonatal domain [11,12,13]. In NICUs, most quality indicators focus on clinical outcomes. More recently, nursing-sensitive indicators have been developed to capture the contribution of nursing care to neonatal outcomes [14]; nonetheless, these indicators exhibit considerable variability and are not consistently integrated into quality monitoring systems [5,6,15].

Feeding difficulties remain one of the principal causes of readmission during the first week after discharge—particularly in preterm infants—driven by neurological immaturity and disordered suck–swallow–breathe coordination [16]. This evidence underscores the importance of safe nutritional transitions between hospital and home and the value of monitoring nutritional care as an indicator of system quality [17,18,19].

Neonatal nurses are the primary implementers of nutrition and feeding interventions [7]; yet their specific contribution to nutritional outcomes remains largely unquantified through validated indicators [20]. Despite this clinical relevance, quality indicators specific to neonatal feeding care are scarce, weakly operationalised and lack consensus [20]. This gap limits comparative evaluation between units and constrains the identification of best practices grounded in evidence [21]. To address this gap, the guiding research question is: in hospitalised neonates, which evidence-based quality indicators, validated through expert consensus, enable systematic evaluation of basic nutrition and feeding care?

## 2. Materials and Methods

### 2.1. Study Design

A two-phase methodological study was conducted, combining a Scoping Review and a modified Delphi study. The Scoping Review followed JBI methodological recommendations [22], was prospectively registered on OSF [23] and reported per PRISMA-ScR [24]. The modified Delphi study was based on the RAND/UCLA Appropriateness Method [25]. The Delphi phase generated expert consensus on the content and operational definition of the indicators and did not constitute empirical validation of their reliability or construct validity; the term “consensus-based” is therefore used throughout.

Nursing-sensitive indicators are understood here as structures, processes and outcomes that are influenced by nursing care and responsive to changes in it, without implying that nursing is exclusively responsible for the result [14]. Indicators were therefore eligible when neonatal nursing influences their performance, even when other professions contribute to the underlying decision—for parenteral nutrition or electrolyte monitoring, for instance, nursing determines the timeliness of initiation, administration, monitoring and documentation. After the Delphi process, each appropriate indicator was classified by its degree of nursing influence as nurse-led, shared or nursing-influenced multidisciplinary (Supplementary Table S7). A panel of neonatal nurses was chosen because nurses deliver and document most bedside feeding care and are best placed to judge operational definitions and the feasibility of routine measurement.

The study was conducted in accordance with the Declaration of Helsinki. Participation was voluntary, anonymous and subject to informed consent (EU Regulation 2016/679; Spanish Organic Law 3/2018). The study was approved by the Research Ethics Committee of Vall d’Hebron Institut de Recerca (protocol code PR(AG)188/2026, date of approval 4 March 2026).

### 2.2. Scoping Review

The review aimed to identify quality indicators related to basic nutrition and feeding care in hospitalised neonates. The research question followed the PCC framework: population (neonates admitted to NICUs or intermediate care units), concept (quality indicators related to basic nutrition and feeding care) and context (hospital neonatal care settings). Four databases—PubMed, Embase, Scopus and the Cochrane Library—were searched on 20 April 2025 (2020–2025), complemented by snowball citation tracking and the grey literature from international institutions. The PubMed strategy combined MeSH terms and free text:

(“Neonatal Nursing”[MeSH] OR “nurses, neonatal”[MeSH] OR “intensive care, neonatal”[MeSH] OR “intensive care units, neonatal”[All Fields]) AND (“models, nursing”[MeSH] OR “patient care management”[MeSH]) AND (“quality of health care”[MeSH] OR “health care quality, access, and evaluation”[MeSH] OR “quality improvement”[MeSH]).

The composition of this strategy was a deliberate methodological decision. In scoping searches conducted while preparing the protocol, combining nutrition- and feeding-specific terms with nursing and quality-indicator terms in a single query proved excessively restrictive and retrieved very little evidence, because sources are rarely indexed under all three concepts simultaneously; conversely, omitting the nursing and care-model terms returned a predominantly clinical corpus in which nursing-sensitive and organisational indicators were difficult to identify. We therefore searched the intersection of neonatal nursing, care models and healthcare quality, applied nutrition and feeding content as an eligibility criterion at the screening stage, and complemented the databases with snowball citation tracking and with internationally established reference documents for neonatal nutrition and feeding care. The 2020–2025 window was applied to capture indicators reflecting current practice and the most recent international standards; earlier documents were eligible only when they remained the current, in-force version of such a framework and were identified through snowballing (File S1).

Sources of any methodological design, including normative documents and clinical guidelines, addressing quality indicators for basic neonatal nutrition or feeding care, were eligible. Studies on non-neonatal populations, exclusively pharmacological or surgical interventions unrelated to basic care, and documents lacking empirical data were excluded. Selection was conducted independently by two reviewers (P.B., E.B.) with discrepancies resolved by a third (D.O.), supported by Rayyan^®^ [26]. Data were extracted using a standardised piloted form capturing source, context, methodology, population, objective, indicator framework and Donabedian dimension. Full eligibility criteria, search strings and results are provided in Supplementary File S1. Records (database hits), reports (full texts assessed), included documents, indicator frameworks (sets of indicators from one programme or organisation, which may span several documents) and source indicators (individual indicators extracted) are distinguished throughout.

Indicator synthesis followed an inductive thematic process: P.B. labelled and grouped indicators by thematic similarity; S.A. synthesised them through combination and minor adaptation; and the team finalised the pre-Delphi set in a focus group. Indicators were operationally defined and forward- and back-translated between English, Spanish and Catalan to ensure conceptual equivalence.

### 2.3. Modified Delphi Study

A panel of 20–25 neonatal nursing professionals was recruited through intentional sampling per RAND/UCLA recommendations [25]; inclusion and exclusion criteria are presented in Supplementary Table S2. Eligibility required at least five years of experience in hospital neonatal care, current employment in a neonatal unit and previous participation in quality, patient safety, guideline or research projects; this last criterion was applied because judging operational definitions and feasibility requires familiarity with indicator construction and data sources, although it may preferentially select professionals interested in quality measurement. Specific expertise in neonatal nutrition was not an eligibility criterion but was recorded at registration through self-rated competence on a 5-point scale. Each indicator was assessed across the following three dimensions using a 9-point Likert scale: appropriateness (degree to which the indicator reflects sound clinical practice), clinical validity (importance for quality and safety of care) and feasibility (possibility of measurement in routine practice), where 1–3 = low, 4–6 = uncertain and 7–9 = high. The three dimensions were rated independently and all contributed to the final classification (Section 2.4). Qualitative comments and modification proposals were permitted. Secondary variables included the following sociodemographic and professional characteristics: age, gender, profession, years of neonatal experience, hospital care level and prior experience in quality or research.

Two rounds were administered via REDCap (VHIR institutional server). In Round 1 (12–26 March 2026), experts rated each indicator and could propose modifications. Indicators lacking consensus in any dimension (interquartile range > 2 points or <75% of panellists rating 7–9) were revised according to the panellists’ comments and re-rated in all three dimensions in Round 2 (16–23 April 2026), in which aggregated results—median, dispersion and qualitative comments—were provided for informed re-evaluation. Two rounds were deemed sufficient per RAND/UCLA stability criteria and to minimise attrition. After the Delphi process, each appropriate indicator was specified with numerator, denominator, eligible population, exclusions and measurement period (Supplementary Table S7); eligible populations were restricted where clinically required, for example, parenteral nutrition within 24 h in infants born at <32 weeks’ gestation or <1500 g. Indicator 8.1 was assessed by the panel as a single, combined morbidity indicator and is therefore retained in this form. Separating its components after the consensus process would result in a construct that was not assessed by the panel. Units are therefore advised to select one component as the primary measure while reporting counts for both components.

### 2.4. Statistical Analysis

Indicators were classified as appropriate (median ≥ 7 AND ≤15% scoring ≤3), uncertain (remaining cases with median > 3) or inappropriate (median ≤ 3) in each dimension; an indicator was classified as appropriate only when all three dimensions met this criterion in its final round. These thresholds are an adaptation of the RAND/UCLA Appropriateness Method [25], which defines disagreement for nine-member panels from the distribution of ratings across the extreme thirds of the scale and proposes the interpercentile range adjusted for symmetry for other panel sizes; with 20–23 panellists, disagreement was operationalised as more than 15% of ratings in the 1–3 range. As this criterion is less conservative, agreement and disagreement percentages are reported for every indicator and round in Supplementary Table S6a so that alternative criteria can be applied [27]. The panel authority coefficient (Cr = arithmetic mean of the competence coefficient Ca and familiarity coefficient Cs, both self-assessed on a 5-point Likert scale normalised to 0–1) was calculated with an acceptability threshold of ≥0.70 [25]. Descriptive statistics were computed in IBM SPSS Statistics v30, with quartiles estimated by linear interpolation; qualitative comments were analysed descriptively.

A post hoc prioritisation analysis was conducted following Multi-Criteria Decision Analysis (MCDA) principles [28], addressing the recognised limitation of binary Delphi consensus classification that provides no guidance on implementation sequencing [27]. It was restricted to the 22 indicators classified as appropriate. Four components, each rescaled to 0–100, were derived from the final-round ratings as follows: *F* (feasibility) = (feasibility median − 1)/8 × 100; *M* (mean rating) = (mean of the three dimension medians − 1)/8 × 100; *A* (agreement) = mean percentage of panellists rating 7–9 across the three dimensions; and *D* (dispersion) = (8 − mean IQR across the three dimensions)/8 × 100, where 8 is the maximum possible IQR on a 1–9 scale. The composite score (*CS*) was calculated as:

*CS* = 0.333 *F* + 0.278 *M* + 0.222 *A* + 0.167 *D*

The weights correspond to the originally planned 30%, 25%, 20% and 15%, renormalised after removing the RAND/UCLA classification component, which was constant across appropriate indicators and could not contribute to their relative ranking. They were set a priori by the research team, giving feasibility the greatest influence because implementability is the main practical constraint on indicator use [25,29], and were not elicited from stakeholders; as feasibility also enters *M*, *A* and *D*, its effective weight is 55.6%. Robustness was examined using equal weights, components computed without feasibility, a feasibility-only score and 10,000 random weight vectors drawn from a flat Dirichlet distribution, comparing rankings with Spearman’s ρ (Supplementary Table S6b). Indicators were grouped into four implementation tiers by rank (1–5, 6–10, 11–15 and 16–22), so that Tiers 1, 1–2 and 1–3 form nested sets of 5, 10 and 15 indicators, consistent with composite neonatal quality index methodology [29]. This analysis was exploratory and not pre-specified; it is presented as a practical decision-support tool, not a validated ranking instrument.

## 3. Results

### 3.1. Scoping Review Findings

**Figure 1:**
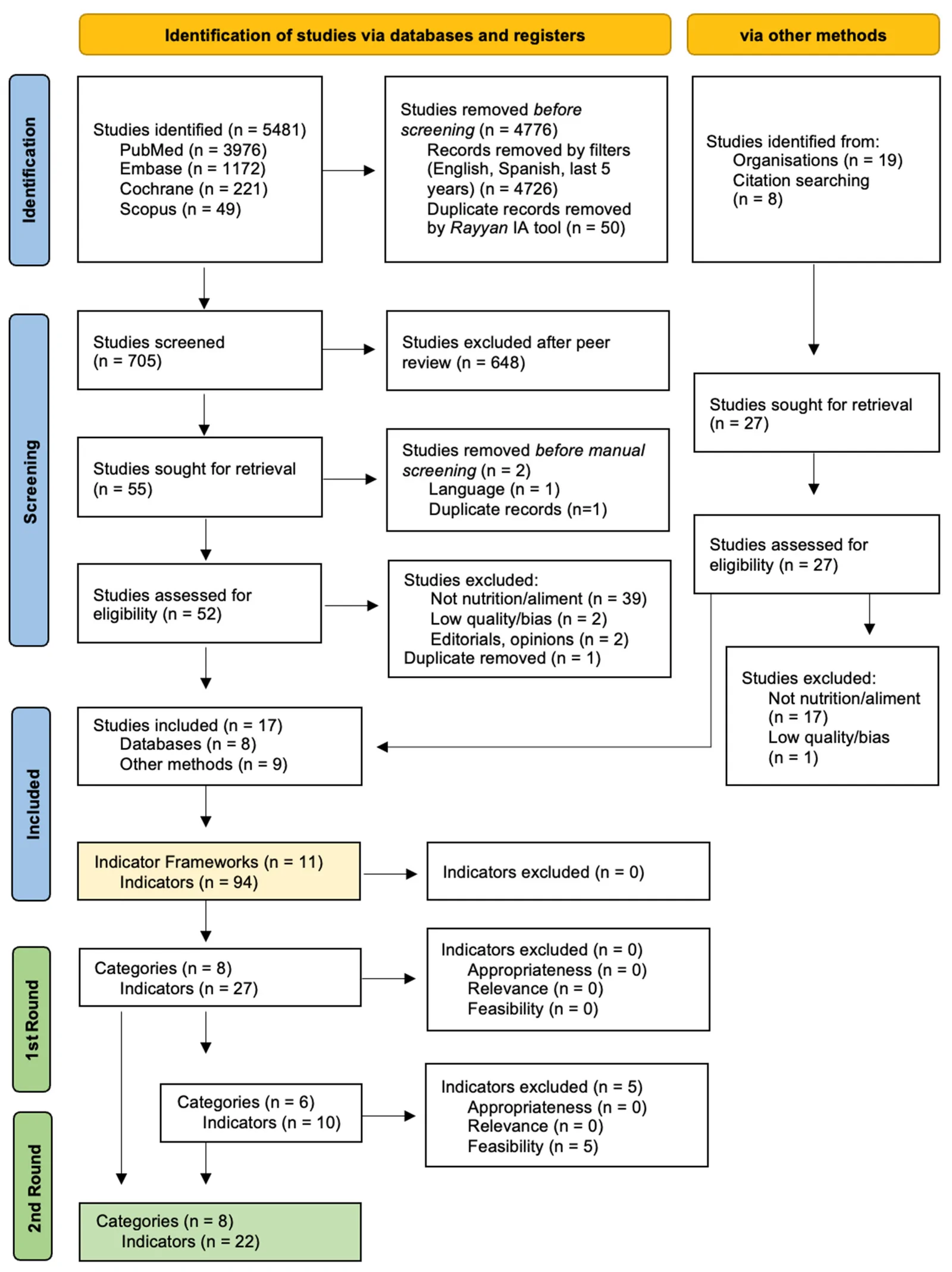
PRISMA-ScR 2020 flow diagram.

The bibliographic search identified 5481 records of which eight met inclusion criteria after deduplication, screening and full-text assessment. A complementary snowball and the grey literature search identified 27 additional sources, of which nine were incorporated, yielding a final corpus of 17 documents (Figure 1). These documents corresponded to 11 indicator frameworks, from which 94 source indicators were extracted (structure, n = 9; process, n = 76; and outcome, n = 9). Documents were published between 2013 and 2025, 14 (82%) from 2020 onwards; the three earlier documents were normative frameworks still in force, retrieved through snowballing. Six documents originated from Canada, four from multinational European consortia, two from the United Kingdom, one each from China, Finland, South Africa and Indonesia, and one was an international society guideline [30,31,32,33,34,35,36,37]. By type, they comprised consensus or Delphi studies (n = 4), study or project protocols (n = 3), clinical guidelines and quality standards (n = 4), implementation or quality-improvement studies (n = 5) and one situational analysis; each document is counted once. Full source characteristics are provided in Supplementary Table S3 and indicators contributed by each source in Supplementary Table S4.

### 3.2. Expert Panel Characteristics

Twenty-three experts participated in Round 1 and twenty in Round 2, yielding a retention rate of 87.0%. The panel was predominantly female (87.0%) with a mean age of 42.3 years (SD 8.7) and mean neonatal experience of 15.3 years (SD 8.3). In terms of professional category, 73.9% were Paediatric Nurse Specialists and the remaining 26.1% were generalist nurses with accredited clinical activity in neonatal care. A total of 47.8% reported previous experience in Delphi processes or quality improvement initiatives. Regarding self-perceived competence in neonatal nutrition, 91.3% of participants considered themselves experts or highly expert (eight highly expert, 34.8%; 13 expert, 56.5%; and two neutral, 8.7%). The mean authority coefficient was 0.84 in Round 1 and 0.83 in Round 2—both exceeding the 0.70 threshold, supporting panel suitability. The three panellists who did not complete Round 2 were comparable to those retained in professional category (two Paediatric Nurse Specialists and one generalist nurse), age (mean 40.0 vs. 42.6 years), neonatal experience (15.3 vs. 15.2 years) and self-rated nutrition expertise (all expert or highly expert), and their Round 1 feasibility ratings for the ten re-evaluated indicators did not differ (mean 6.6 vs. 6.6).

### 3.3. Delphi Results

In Round 1, the overall median for appropriateness was 9.0 (IQR: 0), for clinical validity 9.0 (IQR: 0.5) and for feasibility 9.0 (IQR: 1.8). One hundred percent of indicators (n = 27) achieved median scores ≥ 7 in appropriateness and clinical validity, with agreement ranging from 61% to 100% in both dimensions and from 30% to 100% for feasibility (Supplementary Table S6a), while 10 indicators (37%) presented dispersion or agreement below the RAND/UCLA threshold in the feasibility dimension and were submitted to Round 2. The greatest dispersion was observed in the organisation and governance domain (mean feasibility IQR: 3.8), whereas categories relating to initiation of nutrition and human milk use showed the greatest homogeneity (mean IQR: 0.8).

Modifications between rounds comprised the following two types: terminological clarifications or denominator delimitations without altering the underlying construct (indicators 3.1, 3.2, 6.2 and 7.3), and more substantial revisions affecting the scope, measurement conditions or operationalisation of the indicator (indicators 2.4, 2.5 and 4.1). Full details of all modifications are provided in Supplementary Table S5.

Following Round 2, a mean reduction of 18% in the interquartile range was observed across reviewed indicators. Median variation between rounds was ≤1 point in all ten revised indicators. Final classification yielded 22 indicators (81.5%) as appropriate, five (18.5%) as uncertain and zero as inappropriate (Table 1 and Table 2). The five uncertain indicators met the criteria for appropriateness and clinical validity and failed them only for feasibility.

### 3.4. Consensus Patterns and Domain Distribution

Indicators reaching the highest consensus (median = 9, IQR ≤1) were concentrated in the domains of early nutrition initiation, prioritisation of human milk use and discharge planning. Conversely, indicators of developmental feeding practices (4.1, 4.3) and organisation and governance (6.2) showed greater variability and lower perceived feasibility, remaining uncertain after both rounds, together with indicators 2.4 and 2.5.

The final consensus-based set consisted of 22 indicators classified as appropriate, distributed across the eight conceptual domains. The composition by Donabedian dimension was 72.7% process indicators (n = 16), 13.6% structure indicators (n = 3) and 13.6% outcome indicators (n = 3). By degree of nursing influence, seven indicators were classified as nurse-led, 10 as shared and five as nursing-influenced multidisciplinary (Supplementary Table S7).

### 3.5. Prioritisation Analysis

**Figure 2:**
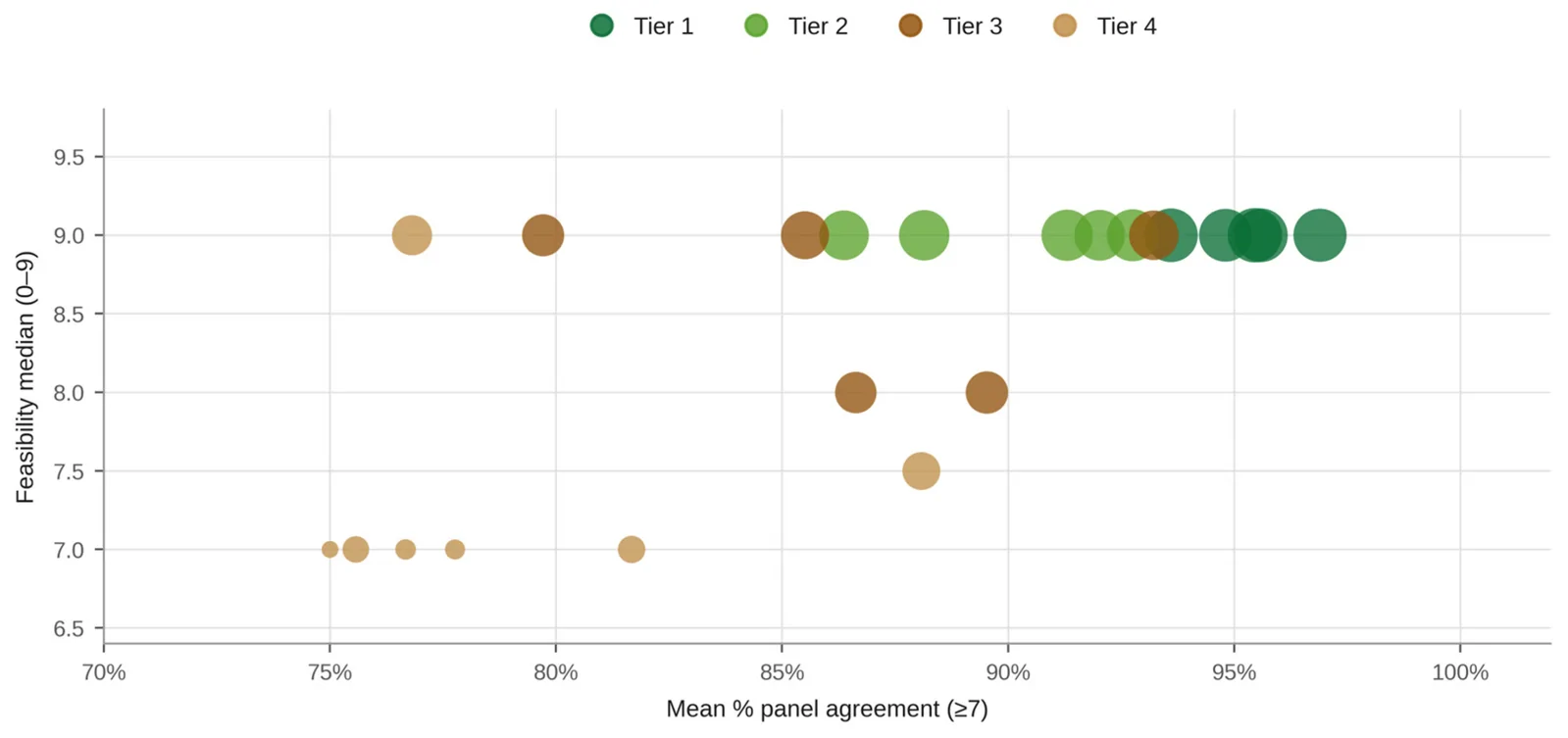
Implementation priority map: feasibility median vs. mean panel agreement, bubble size proportional to composite score.

The post hoc prioritisation analysis was applied exclusively to the 22 appropriate indicators, identifying four implementation tiers (Figure 2; full component values in Supplementary Table S6b). Tier 1 comprised indicators 8.1, 6.3, 2.1, 2.3 and 1.2 (Table 3); Tier 2, indicators 5.1, 1.1, 7.1, 5.3 and 6.1; Tier 3, indicators 1.3, 2.2, 4.2, 6.4 and 7.2; and Tier 4, indicators 5.2, 3.3, 7.3, 3.1, 8.2, 3.2 and 3.4. The five Tier 1 indicators achieved the maximum feasibility median (9.0), an IQR ≤ 1 in every dimension and cover outcome (D8), structure (D6) and process (D1, D2) dimensions. The ranking was robust to the weighting scheme: equal weights (ρ = 0.998) reproduced every tier assignment and the five Tier 1 indicators ranked in the top five in 100% of 10,000 random weightings, whereas removing feasibility from the remaining components changed the ranking more (ρ = 0.967; 16 of 22 indicators retained their tier). Tiers 1–2 together form a basic monitoring dashboard of 10 indicators; Tiers 1–3 configure a standard 15-indicator set covering seven of the eight domains, since all lactation-support indicators (domain 3) fell into Tier 4. Tier 4 indicators, while appropriate, require prior organisational development before systematic measurement is feasible.

## 4. Discussion

This study identified, agreed through expert consensus and prioritised 22 quality indicators for basic nutrition and feeding care in hospitalised neonates, combining a scoping review with a modified RAND/UCLA Delphi. Unlike prior work centred on clinical outcomes or isolated interventions, it integrates structural, process and outcome dimensions within the Donabedian model and adds an explicit nursing-sensitive perspective, addressing a long-standing gap in operationalising neonatal nutrition quality.

The indicators align with NICE [38], the European Standards of Care for Newborn Health [39], particularly regarding the promotion of human milk, early initiation of nutrition and lactation support. Indicators 2.1, 2.3 and 3.3 operationalise these international priorities [40,41]. The continuity-of-care indicators (7.1, 7.2, 7.3) respond to evidence consolidated in the Family Centred Maternity and Newborn Care framework and the National Neonatal Audit Programme [42], which identify the hospital-to-home transition as a moment of nutritional vulnerability where documented discharge planning and community referral are essential for post-discharge safety.

Unlike prior descriptive work or consensus based on unstructured agreement, this study applied a formal appropriateness and consensus procedure following RAND/UCLA criteria [43,44]. The iterative refinement of indicators between rounds—for instance, incorporating the measurement method in indicator 2.4—resolved initial ambiguity, following an approach analogous to Giltenane et al. [45] and van Varsseveld et al. [21]. Nursing sensitivity was applied as an explicit selection criterion, in the sense of care that nursing delivers or influences rather than care for which nursing is solely accountable, in alignment with the nursing-sensitive framework applied in the neonatal context by Chen et al. [14]. Of the 22 appropriate indicators, seven are predominantly nurse-led, 10 reflect shared responsibility and five—including parenteral nutrition initiation, electrolyte monitoring and nutrition-related morbidity—are influenced by nursing but determined mainly by multidisciplinary decisions (Supplementary Table S7); for these, nursing influences the timeliness of initiation, administration, monitoring and documentation rather than the clinical decision itself. Indicators such as 3.3 and 4.2 capture autonomous nursing interventions that are rarely included in standard monitoring systems.

The consensus on indicator 5.1 (human milk fortification per protocol) and 5.3 (safe handling of expressed breast milk) addresses the concern, recognised in the ESCNH standards [39] and the Bliss Baby Charter [46], that nutritional quality depends not only on access to human milk but on its safe administration and optimisation. The set also incorporates organisational and continuity dimensions (6.1, 6.3, and 6.4) inconsistently present in previous frameworks, recognising that quality depends on the structural conditions that make interventions feasible and sustainable.

Five indicators remained uncertain after both rounds (2.4, 2.5, 4.1, 4.3, and 6.2). This uncertainty did not reflect questioning of clinical relevance—these indicators received appropriateness and clinical-validity medians of 7 to 9 in both rounds—but concerns about immediate feasibility where the organisational preconditions are not yet established. This pattern is consistent with critiques that neonatal indicator sets often lack clear operational definitions and rigorous validation, limiting their utility across contexts [14,47]. Conversely, the adapted disagreement threshold is less conservative than the original RAND/UCLA definitions, so indicators reaching consensus with moderate agreement—notably the lactation indicators 3.1, 3.2 and 3.4, with 60% agreement on feasibility in Round 2—should be regarded as provisional.

A key contribution beyond consensus is a structured prioritisation framework to guide implementation across units with different resource levels, addressing a recognised limitation of previous sets [29]. The highest weight assigned to feasibility (30%) reflects the principle that implementability is the primary constraint in indicator selection [25] and is consistent with the actionability-based weighting used in Baby-MONITOR [29]; since feasibility also contributes to the other components, its effective weight was 55.6%. The Tier 1 core set was insensitive to the specific weights chosen, but depended on the emphasis placed on feasibility, which is by design; as the weights were set by the research team rather than elicited from stakeholders, the tiers should be read as hypotheses for implementation research rather than as a validated sequence. The Tier 1 core set—one outcome (NEC/late-onset sepsis), one structure (staff training) and three process indicators (human milk initiation and prioritisation)—provides a minimum viable dashboard requiring only routinely available data. The progression from Tier 1 to Tier 3 mirrors the stepwise approach recommended for NICU quality improvement [29] and the level-stratified framework of Stark et al. [48]. Emerging clinical decision-support platforms confirm that real-time integration of feeding and nutritional indicators is technically feasible [49], supporting progressive digital adoption. Notably, no lactation-support indicator reached Tiers 1–3, reflecting lower perceived feasibility rather than lower clinical relevance and suggesting that data infrastructure for lactation care is an implementation priority in itself.

Once tested, the indicator set could offer a pragmatic tool for clinical practice, education, management and policy. Clinically and operationally, the indicators are intended to support systematic monitoring of nutritional care quality, reduction in clinical variability, internal audits and benchmarking across units, and integration into clinical dashboards and continuous improvement programmes. From a nursing perspective, the set visibilises the specific contribution of neonatal nursing, recognising autonomous practices in lactation support, developmental feeding and parental education as core components of neonatal quality. From an equity standpoint, the focus on basic care captures universal interventions independent of technological level and transferable across resource settings. The following two refinements are already foreseen: separating the two components of indicator 8.1, which the present panel rated jointly, and stratifying denominators by gestational age or birth weight. The planned roadmap is to complete the prioritisation of indicators for each domain of basic neonatal care and then submit the combined set to a broader interdisciplinary panel including families before any clinical dashboard is built.

Several limitations should be acknowledged. First, the panel was composed predominantly of Spanish national experts, which may limit international generalisability; multicentric replication is proposed as a priority future line. Second, the modified Delphi method implies inherent bias linked to structured consensus, and the disagreement criterion was adapted to the panel size. Third, the panel was composed exclusively of nurses, which—while coherent with the nursing-sensitive focus—may limit interdisciplinary perspectives, and prior experience in quality projects was an eligibility criterion, which may have favoured measurement-oriented views. Fourth, the absence of prospective clinical validation means operational performance still requires empirical demonstration. Fifth, the set does not yet link short-term process and outcome indicators with long-term neurodevelopmental outcomes. Sixth, the search strategy deliberately combined nursing, care-model and quality concepts rather than nutrition-specific terms, which may have reduced sensitivity for nutrition-specific indicators, and the prioritisation weights were defined by the research team, with tier boundaries below Tier 2 sensitive to the weighting scheme. Finally, the remote Delphi format limited direct interaction, although it guaranteed anonymity and minimised leadership effects.

## 5. Conclusions

Through structured evidence review and expert consensus, this study identified and prioritised 22 quality indicators for the evaluation of basic nutrition and feeding care in hospitalised neonates. The indicators are clinically relevant, applicable in routine practice and organised within the Donabedian framework. Areas of greater variability—developmental feeding, post-discharge breastfeeding monitoring and governance audits—are identified as priorities for future work. The set is not yet ready for clinical dashboards or benchmarking: once the indicators for each domain of basic neonatal care have been prioritised, the complete set will be submitted to a broader interdisciplinary panel including families and tested prospectively in routine practice. Beyond measurement, its nursing-sensitive perspective visibilises the autonomous role of neonatal nursing and strengthens the alignment between evidence-based practice and patient safety.

## Figures and Tables

**Table 1:** RAND/UCLA results across two Delphi rounds: medians by indicator dimension, agreement and final classification for all 27 candidate indicators.

Code	Indicator	Type	App.	CV	Feas.	R2
**1. Initiation and progression of neonatal nutrition**						
1.1	%neonates on PN < 24 h	P	9	9	9	
1.2	%neonates on EN < 48 h (HM)	P	9	9	9	
1.3	Median days to full EN	P	9	9	9	
**2. Type of feeding and human milk use**						
2.1	%neonates HM as first EN (<48 h)	P	9	9	9	
2.2	%neonates HM during hospitalisation	P	9	9	9	
2.3	%neonates HM at discharge	P	9	9	9	
2.4	%coherence milk type–protocol	P	8	7	6	R2
2.5	%breastfeeding 6 m corrected age	O	9	9	6	R2
**3. Lactation support and milk expression**						
3.1	Median h to first milk expression	P	9	9	7	R2
3.2	%mothers lactogenesis II < 72 h	P	8	8	7	R2
3.3	%mothers lactation support < 48 h	P	9	9	7.5	
3.4	%mothers lactation education 14 d	P	9	8	7	R2
**4. Developmental feeding practices**						
4.1	%cue-based feeding episodes	P	9	9	5	R2
4.2	%non-nutritive sucking	P	9	9	8	
4.3	%tube–oral transition support	P	9	8	6	R2
**5. Nutritional optimisation and safety**						
5.1	%HM with fortification	P	9	9	9	
5.2	%electrolyte monitoring	P	8	8	9	
5.3	%safe milk handling processes	P	9	9	9	
**6. Organisation, resources and governance**						
6.1	Approved nutrition guideline	S	9	9	9	
6.2	Audits/year on guideline compliance	P	7	8	6.5	R2
6.3	%staff trained in 24 m	S	9	9	9	
6.4	Ratio milk expression stations/beds	S	8	8	9	
**7. Discharge planning and continuity of care**						
7.1	%discharges with nutrition plan	P	9	9	9	
7.2	%parents pre-discharge education	P	9	9	8	
7.3	%community referrals	P	9	9	7	R2
**8. Clinical and nutritional outcomes**						
8.1	Incidence NEC ≥ 2 and/or late sepsis	O	9	9	9	
8.2	%feeding-related adverse outcomes	O	8	9	7	R2

Note. App. = appropriateness; CV = clinical validity; Feas. = feasibility; R2 = inclusion in Round 2; S = structure; P = process; O = outcome. PN = parenteral nutrition; EN = enteral nutrition; HM = human milk; NEC = necrotising enterocolitis. Appropriateness and clinical validity values correspond to Round 1; feasibility values correspond to Round 2 for the indicators marked R2 and to Round 1 for the remainder. Medians, interquartile ranges, agreement (percentage rating 7–9) and disagreement (percentage rating 1–3) for every indicator, round and dimension are reported in Supplementary Table S6a, and operational specifications in Supplementary Table S7.

**Table 2:** Round 2 results for the ten re-evaluated indicators: feasibility medians, agreement and final classification.

Code	Indicator	Median	% ≥ 7	ΔR1–R2	Decision
2.4	Milk type coherence with protocol	6	45%	↓ −1	Uncertain
2.5	Breastfeeding at 6 m corrected age	6	30%	↑ +1	Uncertain
3.1	Median hours to first milk expression	7	60%	↓ −1	Appropriate
3.2	Lactogenesis II < 72 h postpartum	7	60%	↓ −1	Appropriate
3.4	Lactation education within 14 d postpartum	7	60%	—	Appropriate
4.1	Cue-based feeding episodes	5	35%	↓ −1	Uncertain
4.3	Tube-to-oral feeding transition	6	45%	↓ −1	Uncertain
6.2	Nutrition guideline audits	6.5	50%	↓ −0.5	Uncertain
7.3	Community referral at discharge	7	75%	—	Appropriate
8.2	Post-discharge feeding adverse outcomes (30 d)	7	70%	↓ −1	Appropriate

Note. Median scored on a 0–9 scale; % ≥ 7 = percentage of experts rating the indicator between 7 and 9; ΔR1–R2: Increment between rounds, ↑ increase or ↓ decrease; RAND/UCLA classification: appropriate = median ≥ 7 AND ≤15% rated ≤3; uncertain = remaining cases with median > 3; inappropriate = median ≤ 3. Values correspond to the feasibility dimension; appropriateness and clinical validity were also re-rated and are reported in Supplementary Table S6a.

**Table 3:** Tier 1 core set: the five highest-ranked appropriate indicators.

Code	Indicator	Domain	Type	Feas. Med (IQR)	Mean% ≥ 7	CS
8.1	Incidence NEC ≥ 2 and/or late sepsis	D8	O	9.0 (0.0)	95.5	99.0
6.3	%staff trained in 24 m	D6	S	9.0 (0.5)	94.2	98.4
2.1	%neonates HM as first EN (<48 h)	D2	P	9.0 (0.5)	95.6	98.3
2.3	%neonates HM at discharge	D2	P	9.0 (1.0)	96.9	97.9
1.2	%neonates on EN <48 h (HM)	D1	P	9.0 (0.0)	94.2	97.7

Note. Feas. Med (IQR) = feasibility median and interquartile range in the final round; mean % = 7 = mean percentage of panellists rating 7–9 across the three dimensions; CS = composite prioritisation score (0–100; Section 2.4). D1 = initiation and progression of neonatal nutrition; D2 = type of feeding and human milk use; D6 = organisation, resources and governance; D8 = clinical and nutritional outcomes. S = structure; P = process; O = outcome. NEC = necrotising enterocolitis; EN = enteral nutrition; HM = human milk.

## Data Availability

Aggregated indicator-level data supporting the reported results, including all values used to calculate the composite scores, are provided in Supplementary Table S6. The data presented in this study are available on request from the corresponding author. The data are not publicly available due to privacy and ethical restrictions, as the dataset contains identifiable information from a limited panel of expert participants who consented to the use of their data for this research project only.

## Supplementary Materials

The following supporting information can be downloaded at https://www.mdpi.com/article/10.3390/nu18193230/s1, File S1: Scoping Review details (PCC framework, search strategies, their methodological justification and eligibility criteria); Table S2: Expert panel inclusion/exclusion criteria; Table S3: Characteristics of included sources; Table S4: Indicators contributed by each source; Table S5: Modifications between Delphi rounds (original vs. revised wording); File S6: RAND/UCLA statistics by round and dimension (Table S6a) and composite-score calculation dataset with sensitivity analyses (Table S6b); File S7: Operational specifications and degree of nursing influence of the 22 appropriate indicators.

## Abbreviations

The following abbreviations are used in this manuscript:

CS	Composite score
EN	Enteral nutrition
ESCNH	European Standards of Care for Newborn Health
HM	Human milk
IQR	Interquartile range
JBI	Joanna Briggs Institute
MCDA	Multi-Criteria Decision Analysis
NEC	Necrotising enterocolitis
NICE	National Institute for Health and Care Excellence
NICU	Neonatal intensive care unit
NSI	Nursing-sensitive indicators
OSF	Open Science Framework
PCC	Population, Concept, Context
PN	Parenteral nutrition
PRISMA-ScR	Preferred Reporting Items for Systematic Reviews and Meta-Analyses Extension for Scoping Reviews
RAND/UCLA	RAND Corporation/University of California Los Angeles Appropriateness Method
RCT	Randomised controlled trial
SD	Standard deviation
