# Supplementary material for: Quality Indicators for Evaluation of Neonatal Basic Care in Nutrition and Feeding: A Scoping Review and Modified Delphi Study
Source: Nutrients. 2026 Sep 30;18(19):3230. doi: 10.3390/nu18193230 (PMC13666701; doi:10.3390/nu18193230)
Supplement: Supplementary file 1 [file nutrients-18-03230-s001.zip › File S1.pdf]

File S1.

Table S1. *Scoping Review Details — PCC Question, Databases and Selection Criteria*

PCC	Description
P (Population)	Neonates hospitalised in neonatal intensive care units (NICUs) or intermediate neonatal care units.
C (Concept)	Quality indicators related to basic nutrition and feeding care in hospitalised neonates, including parenteral nutrition, enteral nutrition, human milk feeding and lactation support.
C (Context)	Hospital neonatal care settings (intensive, intermediate or transitional care units), in both high-income and low- and middle-income countries.

BBDD	Search strategy (results)
PubMed	("Neonatal Nursing"[MeSH Terms] OR "nurses, neonatal"[MeSH Terms] OR "intensive care, neonatal"[MeSH Terms] OR "intensive care units neonatal"[All Fields]) AND ("models, nursing"[MeSH Terms] OR "patient care management"[MeSH Terms]) AND ("quality of health care"[MeSH Terms] OR "health care quality, access, and evaluation"[MeSH Terms] OR "quality improvement"[MeSH Terms]) = 3976
	(Full text, in the last 5 years) = 528
Embase	('newborn nursing'/exp OR 'neonatal nurse'/exp OR 'newborn intensive care'/exp OR 'intensive care units neonatal') AND ('nursing theory'/exp OR 'patient care'/exp) AND ('health care quality'/exp OR 'total quality management'/exp) = 1172
	AND [2020-2025]/py = 176
Scopus	(ALL ('newborn AND nursing'/exp OR 'neonatal AND nurse'/exp OR 'newborn AND intensive AND care'/exp OR 'intensive AND care AND units AND neonatal') AND ALL ('nursing AND theory'/exp OR 'patient AND care'/exp) AND ALL ('health AND care AND quality'/exp OR 'health AND care AND quality'/exp OR 'total AND quality AND management'/exp) = 49
	AND PUBYEAR > 2019 AND PUBYEAR < 2026 = 18
Cochrane	("Neonatal Nursing"[MeSH] OR "Nurses, Neonatal"[MeSH] OR "Intensive Care, Neonatal"[MeSH] OR "intensive care units neonatal") AND ("Models, Nursing"[MeSH] OR "Patient Care Management"[MeSH]") AND ("Quality of Health Care"[MeSH] OR "Health Care Quality, Access, and Evaluation"[MeSH] OR "Quality Improvement"[MeSH]") = 221(3RS + 218 EC)
	2020-2025 = 1 RS y 32 EC

Note. *SR = systematic review; CT = clinical trial. Searches conducted on 20 April 2025. All searches limited to full-text publications, 2020–2025 (except snowball technique and grey literature sources).*

Inclusion criteria	Exclusion criteria
Studies on hospitalised neonates.	Studies in non-neonatal paediatric or community populations.
Research describing, developing, or evaluating quality indicators related to neonatal nutrition or feeding care.	Exclusively medical or pharmacological interventions with no relation to basic nutrition care.
Studies of any design: quantitative, qualitative, mixed methods or reviews.	Letters, editorials or commentaries without empirical data.
Normative documents, clinical guidelines or recognised quality frameworks.	Studies not available in English or Spanish.

Methodological justification of the search strategy and of the publication window

The database strategy combined three concepts — neonatal nursing, nursing care models/patient care management and healthcare quality/quality improvement — and did not include nutrition- and feeding-specific terms. This was a deliberate decision taken by the research team, based on previous experience in this field and on scoping searches conducted while preparing the protocol:

Strategy tested while preparing the protocol	Observed behaviour and decision
Nursing + quality + nutrition/feeding terms combined in one query	Excessively restrictive: very few records retrieved, because sources are rarely indexed simultaneously under all three concepts. Relevant reference frameworks (e.g., European Standards of Care for Newborn Health, Bliss Baby Charter) were not retrieved. Rejected.
Nutrition/feeding + quality terms, without nursing or care-model terms	Predominantly clinical corpus (nutritional interventions and outcomes) in which indicators sensitive to nursing care were difficult to identify, and structural or organisational indicators were largely absent. Rejected.
Nursing + care models + quality terms, with nutrition and feeding applied as an eligibility criterion at screening (strategy adopted)	Balanced corpus containing both clinical and nursing/organisational indicators; nutrition and feeding content was assessed for every record during dual screening. Complemented by snowball citation tracking and by targeted retrieval of internationally established reference documents for neonatal nutrition and feeding care. Adopted.

Publication window. The 2020–2025 limit was applied to the database search to capture indicators that reflect current practice and the most recent international standards. Documents published earlier were eligible only when they remained the current, in-force version of a normative framework in use at the time of the review (NICE QS37, 2013; Public Health Agency of Canada and Bliss Baby Charter, 2017) and were identified through snowballing; no superseded version of any framework was included.

Performance of the adopted strategy. The two nutrition-specific indicator developments published within the search window — breastfeeding quality indicators for very preterm infants (Lu et al.) and the European core indicator set for necrotising enterocolitis (van Varsseveld et al.) — were both retrieved by the database search, as were the ERAS® neonatal recommendations and the ESCNH documents. The potential loss of sensitivity for nutrition-specific indicators is nonetheless acknowledged as a limitation in the main text.
