# Supplementary material for: Quality Indicators for Evaluation of Neonatal Basic Care in Nutrition and Feeding: A Scoping Review and Modified Delphi Study
Source: Nutrients. 2026 Sep 30;18(19):3230. doi: 10.3390/nu18193230 (PMC13666701; doi:10.3390/nu18193230)
Supplement: Supplementary file 1 [file nutrients-18-03230-s001.zip › File S6.pdf]

File S6.

Table S6a. RAND/UCLA statistics by indicator, round and dimension.

Code	Round	N	App. Med (IQR)	App. %≥7 / %≤3	CV Med (IQR)	CV %≥7 / %≤3	Feas. Med (IQR)	Feas. %≥7 / %≤3	Final class.
1.1	R1	23	9.0 (1)	87 / 0	9.0 (1.5)	91 / 0	9.0 (0)	96 / 0	Appropriate
1.2	R1	23	9.0 (0.5)	91 / 0	9.0 (1)	91 / 0	9.0 (0)	100 / 0	Appropriate
1.3	R1	23–22	9.0 (1)	91 / 0	9.0 (2)	96 / 0	9.0 (2)	91 / 0	Appropriate
2.1	R1	22–23	9.0 (0)	95 / 0	9.0 (0.5)	96 / 0	9.0 (0.5)	96 / 0	Appropriate
2.2	R1	23	9.0 (1.5)	91 / 0	9.0 (1.5)	83 / 0	9.0 (1.5)	83 / 0	Appropriate
2.3	R1	21–22	9.0 (0)	100 / 0	9.0 (1)	95 / 0	9.0 (1)	95 / 0	Appropriate
2.4	R1	23	8.0 (2.5)	65 / 9	7.0 (3)	61 / 9	7.0 (3)	57 / 9	
	R2	20	8.0 (3)	60 / 15	7.5 (2.25)	60 / 5	6.0 (4)	45 / 15	Uncertain
2.5	R1	23	9.0 (2.5)	74 / 9	9.0 (2.5)	74 / 13	5.0 (5)	30 / 22	
	R2	20	8.0 (1.25)	100 / 0	8.0 (1.25)	95 / 0	6.0 (3.5)	30 / 25	Uncertain
3.1	R1	22	9.0 (0)	91 / 0	9.0 (1)	96 / 0	8.0 (2.75)	73 / 14	
	R2	20	9.0 (1)	85 / 0	9.0 (1)	85 / 0	7.0 (3)	60 / 10	Appropriate
3.2	R1	23	8.0 (2)	78 / 4	8.0 (2.5)	74 / 4	8.0 (4)	65 / 13	
	R2	20	8.0 (1.25)	85 / 10	8.0 (1)	85 / 10	7.0 (3)	60 / 15	Appropriate
3.3	R1	23–22	9.0 (1)	96 / 0	9.0 (1)	91 / 0	7.5 (2)	77 / 5	Appropriate
3.4	R1	23	9.0 (2)	78 / 0	8.0 (2)	78 / 0	7.0 (2.5)	65 / 4	
	R2	20	8.0 (1.25)	85 / 0	7.5 (1)	80 / 0	7.0 (3)	60 / 5	Appropriate
4.1	R1	23	9.0 (2)	87 / 0	9.0 (2)	78 / 0	6.0 (4)	43 / 22	
	R2	20	8.0 (2)	80 / 15	8.0 (1.25)	85 / 5	5.0 (3.25)	35 / 15	Uncertain
4.2	R1	23–22	9.0 (1)	96 / 0	9.0 (1)	96 / 0	8.0 (2)	77 / 9	Appropriate
4.3	R1	23	9.0 (1.5)	87 / 4	8.0 (2.5)	74 / 0	7.0 (3.5)	61 / 13	

Code	Round	N	App. Med (IQR)	App. %≥7 / %≤3	CV Med (IQR)	CV %≥7 / %≤3	Feas. Med (IQR)	Feas. %≥7 / %≤3	Final class.
	R2	20	8.0 (3)	70 / 5	7.5 (3)	65 / 10	6.0 (4.25)	45 / 30	Uncertain
5.1	R1	23	9.0 (1)	87 / 4	9.0 (1)	96 / 4	9.0 (0)	96 / 4	Appropriate
5.2	R1	23	8.0 (2.5)	74 / 9	8.0 (2)	78 / 9	9.0 (2)	78 / 13	Appropriate
5.3	R1	23–22	9.0 (0.5)	96 / 0	9.0 (1)	87 / 0	9.0 (1)	82 / 5	Appropriate
6.1	R1	22	9.0 (1)	86 / 0	9.0 (0)	82 / 5	9.0 (1.75)	91 / 0	Appropriate
6.2	R1	23	7.0 (3)	61 / 4	8.0 (3)	65 / 4	7.0 (4)	57 / 9	
	R2	20	7.0 (2)	65 / 0	8.0 (1)	84 / 0	6.5 (2)	50 / 10	Uncertain
6.3	R1	23	9.0 (0)	96 / 0	9.0 (0)	96 / 0	9.0 (0.5)	91 / 0	Appropriate
6.4	R1	23	8.0 (2.5)	74 / 0	8.0 (2)	78 / 0	9.0 (1)	87 / 4	Appropriate
7.1	R1	23–22	9.0 (1.5)	96 / 0	9.0 (1)	91 / 0	9.0 (1)	91 / 0	Appropriate
7.2	R1	23–22	9.0 (1)	91 / 0	9.0 (1)	91 / 0	8.0 (2)	77 / 5	Appropriate
7.3	R1	22	9.0 (1)	86 / 0	9.0 (1.75)	82 / 0	7.0 (3.5)	59 / 0	
	R2	20	8.0 (1.25)	90 / 0	8.0 (1)	80 / 0	7.0 (0.5)	75 / 10	Appropriate
8.1	R1	22	9.0 (0)	91 / 5	9.0 (0)	100 / 0	9.0 (0)	95 / 0	Appropriate
8.2	R1	22–23	8.0 (2)	86 / 0	9.0 (2)	87 / 0	8.0 (3)	65 / 9	
	R2	20	8.0 (1.25)	80 / 0	8.0 (1.25)	80 / 0	7.0 (2.5)	70 / 10	Appropriate

Note. Med = median; IQR = interquartile range (quartiles by linear interpolation); %≥7 = agreement; %≤3 = disagreement (percentages). N = number of valid ratings (range across dimensions). Round 1 N = 23 panellists; Round 2 N = 20. Indicators were re-rated in Round 2 when feasibility IQR > 2 or agreement < 75% in Round 1. Classification rule per dimension: appropriate if median ≥ 7 and ≤ 15% of ratings ≤ 3; inappropriate if median ≤ 3; otherwise uncertain. An indicator is appropriate when all three dimensions are appropriate in its final round.

Table S6b. Composite-score components, corrected composite score, tiers and sensitivity analyses (22 appropriate indicators)

Rank	Code	F	M	A	Mean IQR	D	CS	Tier	CS equal weights	CS no feasibility double count	CS feasibility only	Top-5 (%)	Same tier (%)	Median rank (95% interval)
1	8.1	100.0	100.0	95.5	0	100.0	99.0	1	98.9	99.0	99.0	100	100.0	1 (1–3)
2	6.3	100.0	100.0	94.2	0.17	97.9	98.4	1	98.0	99.0	97.0	100	100.0	2 (2–4)
3	2.1	100.0	100.0	95.6	0.33	95.8	98.3	1	97.9	98.5	98.0	100	100.0	3 (2–3)

Rank	Code	F	M	A	Mean IQR	D	CS	Tier	CS equal weights	CS no feasibility double count	CS feasibility only	Top-5 (%)	Same tier (%)	Median rank (95% interval)
4	2.3	100.0	100.0	96.9	0.67	91.7	97.9	1	97.1	98.4	96.9	100	100.0	4 (1–5)
5	1.2	100.0	100.0	94.2	0.5	93.8	97.7	1	97.0	96.5	100.0	100	100.0	5 (4–5)
6	5.1	100.0	100.0	92.8	0.67	91.7	97.0	2	96.1	96.0	99.0	0	100.0	6 (6–6)
7	1.1	100.0	100.0	91.3	0.83	89.6	96.3	2	95.2	95.0	99.0	0	100.0	7 (7–9)
8	7.1	100.0	100.0	92.6	1.17	85.4	95.9	2	94.5	95.9	95.9	0	99.7	8 (7–10)
9	5.3	100.0	100.0	88.1	0.83	89.6	95.6	2	94.4	96.5	93.9	0	99.5	9 (8–10)
10	6.1	100.0	100.0	86.4	0.92	88.5	95.1	2	93.7	95.4	94.3	0	60.6	10 (9–12)
11	1.3	100.0	100.0	92.6	1.67	79.2	94.9	3	92.9	95.4	93.8	0	57.8	11 (8–16)
12	2.2	100.0	100.0	85.5	1.5	81.3	93.7	3	91.7	94.0	93.0	0	98.7	12 (11–15)
13	4.2	87.5	95.8	89.5	1.33	83.3	89.6	3	89.0	92.8	83.1	0	99.5	13 (11–15)
14	6.4	100.0	91.7	79.7	1.83	77.1	89.4	3	87.1	86.5	95.0	0	46.2	16 (13–18)
15	7.2	87.5	95.8	86.6	1.33	83.3	88.9	3	88.3	91.8	83.1	0	77.7	14 (13–16)
16	5.2	100.0	91.7	76.8	2.17	72.9	88.0	4	85.3	86.5	91.0	0	77.7	17 (14–22)
17	3.3	81.3	93.8	88.1	1.33	83.3	86.6	4	86.6	90.2	79.3	0	49.1	15 (13–17)
18	7.3	75.0	83.3	81.7	0.92	88.5	81.1	4	82.1	82.5	78.1	0	93.0	18 (11–19)
19	3.1	75.0	91.7	76.7	1.67	79.2	80.7	4	80.6	86.3	69.6	0	100.0	19 (17–19)
20	8.2	75.0	83.3	76.7	1.67	79.2	78.4	4	78.5	81.1	72.8	0	100.0	20 (19–20)
21	3.2	75.0	83.3	76.7	1.75	78.1	78.2	4	78.3	82.5	69.6	0	100.0	21 (20–21)
22	3.4	75.0	81.3	75.0	1.75	78.1	77.3	4	77.3	81.1	69.6	0	100.0	22 (21–22)

Note. All components use final-round ratings (Round 2 for re-rated indicators). F = (feasibility median − 1)/8 × 100; M = (mean of the three dimension medians − 1)/8 × 100; A = mean percentage rating 7–9 across dimensions; D = (8 − mean IQR)/8 × 100. CS = 0.333 F + 0.278 M + 0.222 A + 0.167 D (original weights 30/25/20/15% renormalised after removing the RAND/UCLA classification term, constant among appropriate indicators). Effective feasibility weight = 55.6%. Tiers by rank: 1–5 (Tier 1), 6–10 (Tier 2), 11–15 (Tier 3), 16–22 (Tier 4). Sensitivity: equal weights (Spearman ρ vs. CS = 0.998; 22/22 same tier); no feasibility double counting — M, A and D computed from appropriateness and clinical validity only (ρ = 0.967; 16/22); feasibility-only components (ρ = 0.924; 14/22); originally reported formula including the classification term and the (9 − IQR)/8 transformation (ρ = 1.000; 22/22). Monte Carlo: 10,000 weight vectors from a flat Dirichlet(1,1,1,1) distribution (seed 2026); Top-5 = % of simulations ranked 1–5; Same tier = % of simulations in which the indicator retained its tier.
