# Supplementary material for: Quality Indicators for Evaluation of Neonatal Basic Care in Nutrition and Feeding: A Scoping Review and Modified Delphi Study
Source: Nutrients. 2026 Sep 30;18(19):3230. doi: 10.3390/nu18193230 (PMC13666701; doi:10.3390/nu18193230)
Supplement: Supplementary file 1 [file nutrients-18-03230-s001.zip › File S7.pdf]

Table S7. Operational specification and degree of nursing influence for the 22 appropriate indicators

Code	Numerator	Denominator / eligible population	Main exclusions	Period	Degree of nursing influence
1.1	Infants who start parenteral nutrition within 24 h of birth	Live-born infants < 32 weeks' GA or < 1500 g admitted within 24 h of birth	Death or transfer < 24 h; documented palliative care plan	Quarterly	Nursing-influenced multidisciplinary
1.2	Infants who receive a first enteral feed (human milk when available) within 48 h of birth or surgery	Infants admitted within 24 h of birth (or post-operatively) with stay ≥ 48 h	Documented contraindication to enteral feeding (e.g., intestinal obstruction, suspected NEC)	Quarterly	Shared
1.3	Median days from birth (or restart after surgery/fasting) to full enteral feeds (unit-defined, e.g., ≥ 150 mL/kg/day)	Infants < 32 weeks' GA or < 1500 g who reach full enteral feeds	Death or transfer before full feeds	Annual	Shared
2.1	Infants whose first enteral feed is mother's own or donor human milk, started ≤ 48 h	As 1.2	As 1.2; maternal contraindication with no donor-milk indication	Quarterly	Shared
2.2	Infants in whom human milk accounts for > 50% of total enteral volume during the stay	Infants receiving enteral feeds for ≥ 7 days	Maternal contraindication with no donor-milk indication	Quarterly	Shared
2.3	Infants receiving > 50% of enteral volume as human milk in the 24 h before discharge	Infants discharged home alive after a stay ≥ 48 h	Transfers to another hospital	Quarterly	Shared
3.1	Median hours from birth to first documented milk expression	Mothers of admitted infants who intend to provide milk and are separated from their infant	Maternal contraindication; mother not intending to lactate	Quarterly	Nurse-led
3.2	Mothers with documented onset of lactogenesis II ≤ 72 h postpartum	As 3.1, with records coordinated with the maternity unit	As 3.1	Quarterly	Shared
3.3	Mothers with documented professional lactation support ≤ 48 h after neonatal admission	As 3.1	As 3.1	Quarterly	Nurse-led
3.4	Mothers with documented structured education and hands-on training ≤ 14 days postpartum	As 3.1, infant stay ≥ 72 h	As 3.1	Quarterly	Nurse-led

Code	Numerator	Denominator / eligible population	Main exclusions	Period	Degree of nursing influence
4.2	Infants with documented non-nutritive sucking during tube feeding or tube-to-oral transition	Infants tube-fed for ≥ 72 h	Oral malformation; clinical contraindication	Quarterly (audit sample)	Nurse-led
5.1	Human-milk-fed infants fortified according to unit protocol	Infants meeting protocol criteria for fortification	Protocol-defined contraindications	Quarterly (audit sample)	Shared
5.2	Infants with electrolyte monitoring and supplementation according to protocol	Infants receiving PN or meeting protocol monitoring criteria	None	Quarterly (audit sample)	Nursing-influenced multidisciplinary
5.3	Audited expressed-milk handling, storage and administration processes compliant with food-safety standards	Processes audited (random sample) or reported non-conformities	None	Quarterly	Nurse-led
6.1	Written neonatal nutrition guideline approved and reviewed within 3 years (yes/no)	Unit	—	Annual	Nursing-influenced multidisciplinary
6.3	Unit professionals with documented training in neonatal nutrition and lactation support ≤ 24 months	Professionals in the unit establishment at census date	Staff employed < 3 months	Annual	Shared
6.4	Functional milk-expression stations (pump, accessories, private space)	Per 10 neonatal beds at census date	—	Annual	Shared
7.1	Discharges with an individualised nutrition and feeding plan in the discharge summary	Infants discharged home after a stay ≥ 48 h	Transfers; deaths	Quarterly	Shared
7.2	Parents with documented feeding education and guidance before discharge	As 7.1	As 7.1	Quarterly	Nurse-led
7.3	Discharges with referral confirmed by the community service and/or follow-up access ≤ 7 days	As 7.1	As 7.1	Quarterly	Nurse-led
8.1	Infants with NEC ≥ stage 2 (modified Bell criteria) and/or culture-confirmed sepsis with onset > 72 h of life. Units are advised to select one of the two components as the primary measure and to report both counts separately alongside the combined figure	Infants < 32 weeks' GA or < 1500 g admitted and surviving > 72 h (late-onset sepsis may alternatively be expressed per 1000 patient-days)	Congenital gastrointestinal anomalies	Annual	Nursing-influenced multidisciplinary

Code	Numerator	Denominator / eligible population	Main exclusions	Period	Degree of nursing influence
8.2	Infants readmitted for a feeding-related cause ≤ 30 days or with documented oral aversion at follow-up	Infants discharged home	Transfers; deaths	Quarterly (30-day follow-up)	Nursing-influenced multidisciplinary

Note. Operational specifications were drafted by the research team after the Delphi process from the final indicator wording and the definitions of the source frameworks (Supplementary Material S4); they have not yet been piloted. Indicator 8.1 was rated by the panel as a single nutrition-related morbidity indicator and is therefore kept as validated; separating its two components would create a construct the panel did not assess, so units are advised to select one component as the primary measure and report both counts. Formal separation is proposed as a refinement for the next validation phase. Degree of nursing influence (classified by the research team after the Delphi process): nurse-led = predominantly within nursing autonomy (n = 7); shared = nursing and other professions jointly accountable (n = 10); nursing-influenced multidisciplinary = nursing influences timeliness of initiation, administration, monitoring and documentation, while the underlying decision or outcome is determined mainly by medical, dietetic or pharmacy decisions or by the team as a whole (n = 5). Consistent with the definition adopted in the manuscript, nursing sensitivity does not imply exclusive nursing responsibility. GA = gestational age; NEC = necrotising enterocolitis; PN = parenteral nutrition.
