# Supplementary material for: Quality Indicators for Evaluation of Neonatal Basic Care in Nutrition and Feeding: A Scoping Review and Modified Delphi Study
Source: Nutrients. 2026 Sep 30;18(19):3230. doi: 10.3390/nu18193230 (PMC13666701; doi:10.3390/nu18193230)
Supplement: Supplementary file 1 [file nutrients-18-03230-s001.zip › Table S2.pdf]

Table S2. *Inclusion and exclusion criteria for expert panel selection.*

Inclusion Criteria	Exclusion Criteria
Nurses with minimum 5 years of experience in hospital neonatal care.	Failure to respond to Round 1 of the Delphi.
Previous participation in quality, patient safety, clinical guideline, or research projects.	Withdrawal from the process before completion.
Active employment in a hospital neonatal unit.	
Voluntary acceptance to participate and availability to complete all Delphi rounds.	
