# Supplementary material for: Quality Indicators for Evaluation of Neonatal Basic Care in Nutrition and Feeding: A Scoping Review and Modified Delphi Study
Source: Nutrients. 2026 Sep 30;18(19):3230. doi: 10.3390/nu18193230 (PMC13666701; doi:10.3390/nu18193230)
Supplement: Supplementary file 1 [file nutrients-18-03230-s001.zip › Table S3.pdf]

Table S3. Scoping Review Results — Included Sources and Indicators Contributed by Type

Source	Context	Type	Population/Panel	Objective	ST	PR	OU
Core Indicator Set for Measuring Quality of Care in Necrotising Enterocolitis: A European Delphi Study [21]	Europe (19 countries)	Modified Delphi method (3 rounds + consensus meeting).	123 initial experts (neonatologists, surgeons, researchers and patient representatives).	Development of a core quality indicator set (NEC Core QI Set) for measuring quality of care in necrotising enterocolitis (NEC).	0	4	0
Using the Delphi Method to Develop Breastfeeding Quality Indicators for Very Preterm Infants [20]	China	Modified Delphi method (2 rounds) based on RAND/UCLA Appropriateness Method. Consensus: DI < 1 and median 7–9.	33 experts (neonatal medicine, neonatal nursing, lactation consultancy). Acceptable expert authority coefficient (Cr).	Development of breastfeeding quality indicators specific to very preterm infants (VPIs).	0	10	1
European Standards of Care for Newborn Health (ESCNH) [31,42,43]	Neonatal units in Europe.	Protocol for the development of a digital self-assessment tool via eDelphi.	At least 50 professionals for the Delphi panel and 20 hospitals for piloting.	Development of a tool to assess the level of compliance with European neonatal care standards (ESCNH).	4	10	3
Comprehensive mapping of NICU developmental care nursing interventions [2]	Neonatal intensive care unit (NICU).	Scoping Review protocol following JBI methodology.	Preterm neonates (<37 weeks) and their parents.	Identify literature on nursing developmental care interventions and propose nursing-sensitive outcome indicators.	0	6	1
Enhanced Recovery After Surgery® for Neonates [44]	NICUs in Canada and the United Kingdom.	Modified Delphi consensus (3 rounds) and targeted literature review.	Advisory panel of surgeons, dietitians, pharmacists, parents and 18 experts.	Develop ERAS recommendations applicable to all surgical neonates in the NICU.	0	4	0
Implementation of the Neonatal ERAS Guideline [6].	NICU, Alberta, Canada.	Mixed-methods study (semi-structured interviews and surveys).	10 neonates, 13 interviewed professionals and 8 survey respondents.	Investigate professionals' perceptions of the facilitation and implementation of the neonatal ERAS guideline.			
Consensus Guidelines: ERAS Society Recommendations [45]	International (ERAS Society).	Evidence-based clinical practice guideline using systematic review and consensus.	Multidisciplinary experts (surgeons, neonatologists, anaesthesiologists) with parental input.	Develop an ERAS guideline to standardise and improve perioperative care for neonates undergoing intestinal surgery.			

Bringing Enhanced Recovery After Surgery to the NICU [46]	Subspecialty NICU, Alberta, Canada.	Implementation trial with retrospective audit of clinical records.	10 infant–parent dyads.	Assess the feasibility and fidelity of implementing the ERAS guideline for intestinal resection.			
Close Collaboration with Parents intervention [7]	Eight neonatal units in Finland.	Mixed-methods pre-post intervention study.	Multiprofessional unit staff and parents (n=26 pre / 36 post).	Evaluate the effects of an educational intervention on the quality of family-centred care (FCC).	2	13	0
Bliss Baby Charter audit tool [38]	Neonatal units in the United Kingdom.	Practical audit framework for self-assessment.	Neonatal unit staff and families.	Standardise high-quality family-centred services to improve outcomes.			
Compliance With Best Practice Guidelines ... [47]	25 NICUs of a private provider in South Africa.	Situational analysis in 2 cycles (structured observations and focus groups).	25 NICUs and regional nursing representatives.	Assess compliance of nursing practices with NDSC best practice guidelines.	0	3	0
Neuroprotection Care Bundle Implementation [48]	Level III NICU, Calgary, Canada.	Quality improvement (QI) initiative with pre-post cohort study.	671 extremely preterm infants (<29 weeks).	Evaluate the impact of a neuroprotective care bundle on the risk of acute brain injury or death.	0	4	0
Reducing neonatal mortality through Clinical Mentorship... [1]	Biak Regional Hospital, Indonesia.	Quality improvement (QI) through intensive clinical mentorship.	Neonatal nursing staff and paediatricians in a low-resource hospital.	Evaluate the impact of a mentorship programme on neonatal mortality.	0	4	0
Family-Centred Maternity and Newborn Care: Breastfeeding [49]	Canada.	National evidence-based guideline and consensus (BFHI adaptation).	Healthcare professionals, families and volunteers in maternity services.	Provide recommendations for the protection and support of breastfeeding.	2	14	3
Postnatal care: Quality standard [QS37] [30]	United Kingdom (England and Wales).	Quality standard based on referenced clinical guidelines.	Parents, infants and postnatal care professionals.	Define priority areas for quality improvement in postnatal care.	1	4	1

Note. S = structure indicator; P = process indicator; O = outcome indicator. n values indicate the number of indicators extracted or adapted per source and dimension after synthesis and deduplication. Sources included via snowball technique or grey literature are not listed separately but are integrated in the 17 included studies. ERAS = Enhanced Recovery After Surgery; ESCNH = European Standards of Care for Newborn Health; FCC = family-centred care; JBI = Joanna Briggs Institute; NDSC = Neurodevelopmental Supportive Care; NEC = necrotising enterocolitis; NICU = neonatal intensive care unit; PHAC = Public Health Agency of Canada; VPI = very preterm infant.
