# Supplementary material for: Quality Indicators for Evaluation of Neonatal Basic Care in Nutrition and Feeding: A Scoping Review and Modified Delphi Study
Source: Nutrients. 2026 Sep 30;18(19):3230. doi: 10.3390/nu18193230 (PMC13666701; doi:10.3390/nu18193230)
Supplement: Supplementary file 1 [file nutrients-18-03230-s001.zip › Table S4.pdf]

Table S4. Included sources and indicators by Donabedian dimension

Author	Indicator
Varsseveld et al., 2025 [21]	• Median day of reinitiation of enteral feeding, including trophic feeds, after nil per mouth upon NEC diagnosis in NEC patients (P)<br>• Median day of reaching full enteral feeding after nil per mouth upon NEC diagnosis in NEC patients (P)<br>• There is a protocol for human (donor) milk administration upon refeeding after NEC (Y/N) (P)<br>• Percentage of NEC patients started on enteral human mother's own milk vs. human donor milk versus artificial formula versus a combination (specify components and more or less than 50% human) upon refeeding after NEC (P)
Lu, 2025 [20]	• Exclusive mother's own milk (MOM) in the 24 hr before discharge (O)<br>• Time of first milk expression (hours after birth) (in *Mother's Lactation Status Indicators*) (P)<br>• Time of Lactogenesis II onset (hours after birth) (in *Mother's Lactation Status Indicators*) (P)<br>• Lactation establishment (in *Mother's Lactation Status Indicators*) (P)<br>• The first time of mother's own milk feeding (P)<br>• Oropharyngeal administration of colostrum (P)<br>• Standardized fortification of human milk (P)<br>• Necrotizing Enterocolitis (≥ stage 2) (in *Balancing Indicators*) (P)<br>• Late-onset sepsis (in *Balancing Indicators*) (P)<br>• Growth velocity during hospitalization (in *Balancing Indicators*) (P)<br>• The time to reach full enteral feeds (in *Balancing Indicators*) (P)
ESCNH [31,42,43]	• Percentage of very preterm infants (<32 weeks of gestation) or very low birth weight infants (<1500 g) who initiate parenteral nutrition within the first 24 hours of life (*Standard 1* adaptation) (P)<br>• Availability of standardized parenteral nutrition solutions 24 hours a day, 7 days a week in the neonatal unit (*Standard 1* adapt.) (S)<br>• Percentage of preterm infants in whom enteral feeding is initiated within the first 48 hours after birth (*Standard 2* adapt.) (P)<br>• Percentage of preterm infants who receive mother's own milk as the first choice for enteral feeding (*Standard 2* adapt.) (P)<br>• Percentage of parents who receive structured education and training on feeding their preterm infant before hospital discharge (*Standard 3* adapt.) (P)<br>• Percentage of late preterm infants (34–36 weeks of gestation) with an individualized and documented nutritional care plan prior to discharge (*Standard 4* adapt.) (P)<br>• Rate of hospital readmission related to feeding or nutritional problems within 30 days after discharge in late preterm infants (*Standard 4* adapt.) (O)<br>• Percentage of infants with complete and regular documentation of weight, length, and head circumference measurements during hospitalisation (Standard 5 adapt.) (O)<br>• Use of agreed and appropriate growth charts for preterm and ill infants in the neonatal unit (*Standard 5* adapt.) (S)<br>• Percentage of preterm infants receiving mother's own milk, either exclusively or partially, at the time of hospital discharge (*Standard 6* adapt.) (O)<br>• Median time from birth to first milk expression by mothers of preterm infants (*Standard 6* adapt.) (P)

	• Percentage of preterm infants without access to mother’s own milk who receive donor human milk instead of formula (*Standard 7* adapt.) (P)<br>• Percentage of preterm infants receiving appropriate human milk fortification according to unit nutritional guidelines (*Standard 8* adapt.) (P)<br>• Percentage of preterm infants who receive preterm formula when mother’s own milk and donor human milk are not available or not indicated (*Standard 9* adapt.) (P)<br>• Availability of a written, evidence-based neonatal nutrition guideline that is regularly reviewed and updated (*Standard 10* adapt.) (S)<br>• Percentage of healthcare professionals working in the neonatal unit who have received formal training on the current neonatal nutrition guideline (*Standard 10* adapt.) (P)<br>• Existence of regular audits of neonatal nutritional practices with documented feedback and quality improvement actions (*Standard 11* adapt.) (S)
Héon, 2022 [2]	• Proportion of preterm infants who initiate trophic enteral feeding with human milk (*Early feeds [trophic, donor milk]* adapt.) (P)<br>• Percentage of feeding episodes conducted using a cue-based approach, with nursing documentation of infant readiness, stress cues, and self-regulation during feeding (*Cue-based feeding* adapt.) (P)<br>• Proportion of eligible preterm infants who receive nurse-led non-nutritive sucking interventions during procedures or prior to oral feeding attempts (*Non-nutritive sucking* adapt.) (P)<br>• Percentage of oral feeding episodes adapted to infant behavioural cues (*Behaviour-based and baby-led feeding* adapt.) (P)<br>• Proportion of mothers of preterm infants who receive structured and documented nursing support for breastfeeding during hospitalisation (*Breastfeeding promotion and support* adapt.) (P)<br>• Proportion of days in which the preterm infant meets estimated nutritional requirements (*Optimisation of nutrition* adapt.) (P)<br>• Incidence of signs of oral aversion among preterm infants (*Nursing interventions optimising directly or indirectly oral human milk feeding of the preterm infant* adapt.) (O)
ERAS [6,44–46]	• Percentage of neonates in whom enteral feeding is attempted within 48 hours after surgery or once clinical eligibility is achieved (P)<br>• Percentage of neonates receiving human breast milk (mother’s own or donor milk) as the first enteral feed after surgery (P)<br>• Percentage of neonates with an ileostomy in whom urinary sodium is measured after surgery according to the unit protocol (P)<br>• Percentage of neonates with an ileostomy in whom mucous fistula refeeding is implemented with the aim of improving growth (P)
Bliss [7,38]	• Number of documented audits per year assessing compliance with the unit’s infant feeding guideline (*Standard 6.1* adapt.) (P)<br>• Percentage of mothers of preterm infants who receive practical support to initiate breast milk expression within the first 2–6 hours after birth (*Standard 6.1* adapt.) (P)<br>• Percentage of mothers who receive ongoing, individualised support to establish and maintain lactation throughout the neonatal stay (*Standard 6.1* adapt.) (P)<br>• Percentage of mothers instructed in evidence-based techniques to optimise milk production within the first 10-14 days postpartum (*Standard 6.1* adapt.) (P)<br>• Percentage of parents who receive clear and consistent information on the benefits of breast milk and the importance of frequent milk expression (*Standard 6.1* adapt.) (P)<br>• Percentage of neonatal staff who receive training on the benefits of breast milk, lactation physiology and practical breastfeeding support (*Standard 6.1* adapt.) (P)<br>• Ratio of breast milk expression devices to mothers of infants admitted to the neonatal unit (*Standard 6.1* adapt.) (S)

	• Number of reported non-compliance events related to breast milk handling, storage or transport protocols per 100 neonatal admissions (*Standard 6.1* adapt.) (P)<br>• Number of private breast milk expression spaces available per 10 neonatal beds (*Standard 6.1* adapt.) (S)<br>• Number of eligible preterm infants receiving breast milk fortification according to the unit's policy (*Standard 6.1* adapt.) (P)<br>• Percentage of parents receiving timely support to facilitate transition from tube feeding to breastfeeding, including recognition of feeding cues and positioning (*Standard 6.2* adapt.) (P)<br>• Percentage of mothers supported to establish breastfeeding prior to neonatal unit discharge (*Standard 6.2* adapt.) (P)<br>• Percentage of neonatal discharges in which breastfeeding or milk expression is documented in the discharge plan (*Standard 6.2* adapt.) (P)<br>• Number of neonates eligible for donor human milk who receive donor human milk in accordance with the unit's protocol (*Standard 6.3* adapt.) (P)<br>• Number of feeding episodes using preterm formula or nutritional supplements that are compliant with the unit's written policy (*Standard 6.3* adapt.) (P)
Rheeder, 2017 [47]	• Proportion of preterm infants receiving non-nutritive sucking during gavage feeding to support transition to oral feeding (P)<br>• Proportion of enteral feeds using mother's own milk or expressed human milk rather than formula (P)<br>• Proportion of neonatal staff trained in neurodevelopmental supportive feeding practices (P)
Murthy, 2020 [48]	• Percentage of infants who receive parenteral nutrition within the first 24 hours of life, including intravenous lipid supplementation (P)<br>• Percentage of infants who initiate enteral feeding within the first 48 hours of life using mother's own milk or donor human milk (P)<br>• Percentage of infants receiving human milk (mother's own or donor) as the first enteral feed (P)<br>• Percentage of infants receiving sodium acetate supplementation as part of parenteral nutrition or arterial line fluids to prevent metabolic acidosis (P)
Kresnawati, 2025 [1]	• Percentage of neonatal unit staff who complete structured lactation training and successfully demonstrate manual breast milk expression (P)<br>• Number of trained lactation counsellors available per neonatal unit following completion of a certified 40-hour WHO–UNICEF–based training programme (P)<br>• Percentage of expressed breast milk handling, storage and preparation processes that comply with food safety standards within the neonatal unit (P)<br>• Percentage of low-birth-weight infants who receive kangaroo mother care during NICU admission, supported by the availability of maternal accommodation (P)
NICE, 2022 [30]	• Percentage of parents of infants admitted to the neonatal unit who receive personalised information and advice about feeding during hospitalisation or prior to discharge (*Statement 2* adapt) (P)<br>• Percentage of infants in the neonatal unit whose feeding is directly observed and assessed by a healthcare professional within 24 hours of admission or initiation of oral feeding (*Statement 4* adapt) (P)<br>• Percentage of neonatal healthcare professionals who have received documented training in breastfeeding and neonatal feeding support (*Statement 4* adapt) (S)<br>• Percentage of neonatal discharge summaries that include a documented feeding and nutrition plan (breastfeeding, expressed milk, donor milk or formula) (*Statement 2 and 4* adapt) (P)

	• Rate of unplanned hospital readmissions or clinical complications related to feeding or nutrition following discharge from the neonatal unit (*Statement 2* adapt) (O)<br>• Percentage of parents of infants with prolonged neonatal admission who receive guidance on age-appropriate nutritional progression prior to discharge (*Statement 7* adapt) (P)
PHAC, 2019[49]	• Percentage of infants exclusively breastfed for the first 6 months of life (O)<br>• Percentage of infants who continue breastfeeding at 12 and 24 months with appropriate complementary feeding (O)<br>• Degree of compliance with the International Code of Marketing of Breast-milk Substitutes, assessed through periodic audits (*Step 1* adapt) (S)<br>• Percentage of staff, healthcare providers and volunteers who acknowledge receipt and understanding of the written infant feeding policy (*Step 1* adapt) (P)<br>• Existence and regular use of a breastfeeding monitoring and data-management system reporting key indicators at least annually (*Step 1* adapt) (S)<br>• Percentage of staff, healthcare providers and volunteers who have completed formal training in breastfeeding support and infant feeding policy implementation (*Step 2* adapt) (P)<br>• Percentage of pregnant women and families who receive documented antenatal education on the importance and management of breastfeeding (*Step 3* adapt) (P)<br>• Percentage of mother–infant dyads who experience uninterrupted skin-to-skin contact immediately after birth for at least one hour or until completion of the first feed (*Step 4* adapt) (P)<br>• Percentage of infants who initiate breastfeeding within the first hour after birth (*Step 4* adapt) (P)<br>• Percentage of mothers who receive documented professional support to initiate and maintain breastfeeding during hospitalisation (*Step 5* adapt) (P)<br>• Percentage of mothers receiving targeted support to manage breastfeeding difficulties (e.g. delayed lactogenesis, prematurity, maternal–infant separation) (*Step 5* adapt) (P)<br>• Percentage of mothers supported to exclusively breastfeed during hospitalisation (*Step 6* adapt) (P)<br>• Percentage of mother–infant dyads practising 24-hour rooming-in during hospital stay (*Step 7* adapt) (P)<br>• Percentage of mothers who receive education and support on recognising and responding to infant feeding cues (*Step 8* adapt) (P)<br>• Percentage of feeding episodes documented as responsive or cue-based feeding (*Step 8* adapt) (P)<br>• Percentage of breastfeeding infants not exposed to bottles, teats or pacifiers during hospitalisation (*Step 9* adapt) (P)<br>• Percentage of mothers who receive counselling on the risks associated with artificial teats and pacifiers (*Step 9* adapt) (P)<br>• Percentage of neonatal or maternity discharges with documented referral to community breastfeeding support services or peer-support programmes (*Step 10* adapt) (P)<br>• Percentage of mothers who report having timely access to breastfeeding support within the first week after discharge (*Step 10* adapt) (O)

Note. S = structure indicator; P = process indicator; O = outcome indicator.
