# Supplementary material for: Quality Indicators for Evaluation of Neonatal Basic Care in Nutrition and Feeding: A Scoping Review and Modified Delphi Study
Source: Nutrients. 2026 Sep 30;18(19):3230. doi: 10.3390/nu18193230 (PMC13666701; doi:10.3390/nu18193230)
Supplement: Supplementary file 1 [file nutrients-18-03230-s001.zip › Table S5.pdf]

Table S5. Validated Indicator Set - Final Classification after Two Delphi Rounds

**Indicator (type) (reference)**	**Decision**
1. Initiation and progression of neonatal nutrition	
1.1. Percentage of neonates who initiate parenteral nutrition within the first 24 hours of life, according to clinical indication (P) [31,42,43,48]	Appropriate (R1)
1.2. Percentage of neonates who initiate enteral feeding within the first 48 hours after birth or surgery, using human milk when available (P) [2,6,31,42–46,48]	Appropriate (R1)
1.3. Median number of days required to achieve full enteral feeding after birth, surgery, or a fasting period (P) [20,21]	Appropriate (R1)
2. Type of feeding and use of human milk	
2.1. Percentage of neonates who receive mother's own milk or donor human milk as the first enteral feed, initiated within the first 48 hours after birth or surgery (P) [6,20,31,42–46,48]	Appropriate (R1)
2.2. Percentage of neonates whose predominant enteral nutrition during hospitalisation consists of human milk (mother's own or donor), rather than formula (P) [47,49]	Appropriate (R1)
2.3. Percentage of neonates whose predominant enteral nutrition at hospital discharge consists of human milk (mother's own or donor), rather than formula (P) [20,31,42,43]	Appropriate (R1)
2.4. Percentage of neonates in whom the type of milk provided (mother's own milk, donor human milk, or formula) is consistent with the documented clinical indication and unit protocol, *assessed through audit of clinical records or chart review in a representative sample* (P) [7,21,31,38,42,43]	Uncertain (R1+R2) *
2.5. Percentage of *preterm* neonates *discharged from the NICU* who continue any breastfeeding at 6 months of corrected age after hospital discharge, *according to follow-up clinic data or regional/national registry records* (P) [49]	Uncertain (R1+R2) *
3. Lactation support and breast milk expression	
3.1. Median number of hours from birth to first breast milk expression among mothers of neonates admitted to the neonatal unit, *via manual or mechanical expression, recorded in the neonatal clinical record* (P) [7,20,31,38,42,43]	Appropriate (R1+R2) *
3.2. Percentage of mothers of neonates admitted to the neonatal unit who achieve Lactogenesis II within the first 72 hours postpartum, *according to records coordinated with the maternity or puerperal unit* (P) [20]	Appropriate (R1+R2) *
3.3. Percentage of mothers who receive documented professional lactation support within the first 48 hours after neonatal admission (P) [2,7,30,38,49]	Appropriate (R1)
3.4. Percentage of mothers who receive structured education and hands-on training in breastfeeding and milk expression techniques within the first 14 days postpartum, *with documented evidence of the session in the clinical record* (P) [1,7,31,38,42,43,49]	Appropriate (R1+R2) *
4. Developmental feeding practices	
4.1. Percentage of enteral or oral feeding episodes performed using a cue-based feeding approach, *in units with an implemented standardised assessment tool or scale for this purpose (P)* [1,2,30,49]	Uncertain (R1+R2) *
4.2. Percentage of neonates who receive non-nutritive sucking interventions during tube feeding or during the transition from tube to oral feeding (P) [2,47,49]	Appropriate (R1)

4.3. Percentage of neonates who receive structured support for progressive transition from tube feeding to breastfeeding or oral feeding, including parent involvement and avoidance of artificial teats or pacifiers *when possible* (P) [7,38,49]	Uncertain (R1+R2) *
5. Nutritional optimisation and safety	
5.1. Percentage of neonates receiving human milk who are provided with breast milk fortification in accordance with established nutritional protocols (P) [7,20,31,38,42,43]	Appropriate (R1)
5.2. Percentage of neonates in whom electrolyte monitoring and supplementation are performed according to clinical indication and protocol to prevent metabolic disturbances (P) [6,44–46,48]	Appropriate (R1)
5.3. Percentage of expressed breast milk handling, storage, and administration processes that comply with food safety standards, as assessed through documented audits or non-compliance reporting (P) [1,7,38]	Appropriate (R1)
6. Organisation, resources, and governance	
6.1. Existence of a written neonatal nutrition and infant feeding guideline formally approved and reviewed within the last 3 years (S) [7,31,38,42,43]	Appropriate (R1)
6.2. Number of documented audits conducted per year to assess compliance with neonatal nutrition or infant feeding guidelines*, including process audits, record reviews, or formalised internal evaluations* (P) [7,31,38,42,43,49]	Uncertain (R1+R2) *
6.3. Percentage of neonatal healthcare professionals who have completed documented training in neonatal nutrition and breastfeeding support within the previous 24 months (S) [1,7,30,31,38,42,43,47,49]	Appropriate (R1)
6.4. Ratio of functional breast milk expression stations (pump, accessories, and private space) per 10 neonatal beds (S) [7,38]	Appropriate (R1)
7. Discharge planning and continuity of care	
7.1. Percentage of neonates discharged from the neonatal unit with a documented individualised nutrition and feeding plan included in the discharge summary (P) [7,30,31,38,42,43]	Appropriate (R1)
7.2. Percentage of parents who receive documented education and guidance on feeding, breastfeeding or milk expression, and expected nutritional progression prior to neonatal discharge (P) [7,30,38]	Appropriate (R1)
7.3. Percentage of neonatal discharges with documented referral to community breastfeeding or nutrition support services*, with confirmation of receipt or acceptance by the receiving service* and/or documented access to follow-up care within the first 7 days after discharge (P) [49]	Appropriate (R1+R2) *
8. Associated clinical and nutritional outcomes	
8.1. Incidence of nutrition-related morbidity during neonatal hospitalisation, defined as the occurrence of necrotising enterocolitis (≥ stage 2) and/or late-onset sepsis (R) [20]	Appropriate (R1)
8.2. Percentage of neonates experiencing feeding-related adverse outcomes after discharge, including feeding-related hospital readmission within 30 days and/or documented oral aversion, *according to follow-up clinic data or readmission records* (R) [2,30,31,42,43]	Appropriate (R1+R2) *

Note. Type: S = structure indicator; P = process indicator; O = outcome indicator (Donabedian, 1988). Classification based on RAND/UCLA Appropriateness Method: Appropriate = median ≥7 AND ≤15% of panellists scored ≤3; Uncertain = all other cases with median >3; Inappropriate = median ≤3. (R1) = final classification obtained in Round 1; (R1+R2) = indicator re-evaluated in Round 2 and final classification reflects Round 2 results. Asterisk (*) denotes indicators whose wording was revised for Round 2 based on qualitative feedback from Round 1. NICU = neonatal intensive care unit; HM = human milk; NEC = necrotising enterocolitis; NOMAS = Neonatal Oral-Motor Assessment Scale; EFS = Early Feeding Skills Assessment; SOFFI = Supporting Oral Feeding in Fragile Infants.
